# Incidental Rotator Cuff Abnormalities on Magnetic Resonance Imaging

**DOI:** 10.1001/jamainternmed.2025.7903

**Published:** 2026-02-16

**Authors:** Thomas Ibounig, Teppo L. N. Järvinen, Saara Raatikainen, Tommi Härkänen, Niko Sillanpää, Frank Bensch, Ville Haapamäki, Pirjo Toivonen, Robert Björkenheim, Anssi Ryösä, Kari Kanto, Vesa Lepola, Antti Joukainen, Mika Paavola, Seppo Koskinen, Lasse Rämö, Rachelle Buchbinder, Simo Taimela

**Affiliations:** 1 Finnish Centre for Evidence-Based Orthopaedics, University of Helsinki, Helsinki, Finland; 2 Department of Orthopaedics and Traumatology, Helsinki University Hospital, Helsinki, Finland; 3 Department of Public Health, Finnish Institute for Health and Welfare (THL), Helsinki, Finland; 4 Department of Radiology, HUS Diagnostic Center, Helsinki University Hospital and University of Helsinki, Helsinki, Finland; 5 Department of Orthopaedics and Traumatology, Turku University Hospital, Turku, Finland; 6 Musculoskeletal Health and Wiser Health Care Units, School of Public Health and Preventive Medicine, Monash University, Melbourne, Victoria, Australia

## Abstract

**Importance:**

Shoulder pain is a common musculoskeletal complaint often attributed to rotator cuff (RC) abnormalities. Diagnostic imaging is frequently used, but the association between RC abnormalities and shoulder symptoms remains uncertain.

**Objective:**

To determine the prevalence of RC abnormalities in a general population sample and their association with shoulder symptoms.

**Design, Setting, and Participants:**

Population-based cross-sectional study in a nationally representative random sample of adults aged 41 to 76 years who underwent standardized clinical assessment and bilateral 3-Tesla magnetic resonance imaging (MRI) of the shoulders conducted from February 2023 to April 2024 in Finland. Eligibility criteria included ability to undergo MRI and absence of previous shoulder replacement surgery.

**Exposures:**

Structured interviews, standardized questionnaires, clinical shoulder tests, and shoulder MRI.

**Main Outcomes and Measures:**

RC tendon status was classified on MRI as normal, tendinopathic, partial-thickness tear (PTT), or full-thickness tear (FTT). Shoulder symptoms were defined as pain or dysfunction in the preceding week. The prevalence of RC abnormalities was compared across age groups and between symptomatic and asymptomatic shoulders, adjusting for demographic factors, concurrent MRI findings, and clinical examination.

**Results:**

Among 602 participants (median age, 58 [range, 41-76] years; 52% female), RC abnormalities on MRI were found in 595 (98.7%; 95% CI, 97.5%-99.5%): 25% tendinopathy, 62% PTT, and 11% FTT. The prevalence and severity of abnormalities increased with age but did not differ between sexes. RC abnormalities were present in 96% of asymptomatic shoulders (1039 of 1076) and 98% of symptomatic shoulders (126 of 128). Only FTTs were more prevalent in symptomatic shoulders (14.6%) than in asymptomatic shoulders (6.5%), but this difference diminished after adjustment (absolute difference, 0.8%; 95% CI, −3.4% to 6.0%).

**Conclusions and Relevance:**

In this population-based study, RC abnormalities were nearly universal after age 40 years and showed poor concordance with shoulder symptoms. These findings suggest that RC abnormalities often represent normal age-related changes rather than disease and call into question the clinical value of routine imaging for atraumatic shoulder pain.

## Key Points

**Question:**

What is the prevalence of rotator cuff (RC) abnormalities in the general Finnish population and how do these findings correspond with shoulder symptoms?

**Findings:**

In this cross-sectional study of 602 Finnish adults aged 41 to 76 years who underwent bilateral 3-Tesla shoulder magnetic resonance imaging and clinical assessment, 99% had at least 1 RC abnormality. RC abnormalities were present in both asymptomatic (96%) and symptomatic (98%) shoulders.

**Meaning:**

The findings of this study suggest that RC abnormalities are nearly universal after age 40 years and that routine imaging should not guide diagnosis or treatment of atraumatic shoulder pain.

## Teaser

This cross-sectional study assesses the prevalence of shoulder rotator cuff abnormalities in a general Finnish population and whether diagnostic imaging should guide diagnosis or treatment of atraumatic shoulder pain.

## Introduction

Shoulder pain affects approximately 18% to 31% of the general population globally each month^1,2,3^ and ranks as the third most common musculoskeletal complaint in primary care.^4,5^ Among individuals presenting with shoulder pain, rotator cuff (RC) abnormalities account for up to 85% of cases.^6,7^ Despite a lack of evidence supporting routine use,^8,9,10^ diagnostic imaging is used in approximately 50% of initial evaluations by general practitioners^8^ and considered essential by up to 82% of general practitioners and 56% of specialists.^5,11^ Imaging frequently reveals RC abnormalities, such as tendinopathy or partial-thickness tears (PTTs) and full-thickness tears (FTTs). Management of these structural findings has become increasingly common and contributes to the growing burden on already strained health care systems. In Australia, up to three-quarters of physicians refer patients with shoulder pain to physical therapy, and the use of imaging-guided injections has increased by as much as 46-fold from the year 2000.^11,12,13^ Meanwhile, surgical RC repairs have increased by approximately 2-fold to 7-fold in other high-income countries.^14,15,16^

Yet, emerging evidence from other musculoskeletal regions suggests that the association between imaging abnormalities and clinical symptoms may be less certain than previously assumed.^17,18^ Interpreting the results of diagnostic tests requires an understanding of the baseline prevalence of positive and negative findings in the general population, as well as the association between positive results and symptoms.^19^ Previous systematic reviews on shoulder imaging abnormalities found low to very low certainty evidence regarding their population prevalence and only poor concordance between imaging findings and symptoms.^20,21,22,23,24^ This underscores the need for robust, population-based data. The Finnish Imaging of Shoulder (FIMAGE) study was designed to assess the prevalence of RC abnormalities detected by magnetic resonance imaging (MRI) in the general population and to evaluate the association of these abnormalities with shoulder symptoms.

## Methods

The FIMAGE study was conducted in Finland from February 2023 to April 2024. The protocol was approved by the Helsinki University Hospital Ethics Committee and a reformatted version was published elsewhere.^25^ The study followed the Declaration of Helsinki principles, and all participants provided written informed consent prior to enrollment. Reporting adhered to the Strengthening the Reporting of Observational Studies in Epidemiology (STROBE) reporting guideline.

### Study Design, Setting, and Participants

This cross-sectional observational study included participants from the Health 2000 survey, a nationally representative, longitudinal health study conducted by the Finnish National Public Health Institute. A total of 9922 persons aged 18 years or older were sampled using a nationally representative 2-stage stratified cluster sample in 2000. Data collection included health interviews, questionnaires, a comprehensive health examination, and incorporated data from national health registries.^26,27^ The Health 2000 cohort and its follow-up survey in 2011 have been continuously monitored and remain a valuable epidemiological resource. They are described in more detail in the eMethods in Supplement 1.

In 2022, the Health 2000 database was reviewed to identify all individuals eligible for the FIMAGE study. To be eligible, participants had to meet the following criteria: (1) prior participation in the Health 2000 survey, (2) valid consent for the Health 2000 follow-up, (3) ability to communicate in Finnish or Swedish, (4) ambulatory status, (5) maximum age of 75 years at the time of sampling, and (6) residence within the catchment areas of the 5 university hospitals, ensuring reasonable access to a 3-Tesla (3T) MRI facility. A total of 2368 individuals fulfilling the inclusion criteria were identified. The youngest participants of the Health 2000 survey were 40 years old by the sampling date of December 1, 2022. A random sample (1284) was invited to participate in the FIMAGE study via informational letters. We confirmed the comparability of our invited sample to the original Health 2000 cohort using national registry data, observing no substantial differences in key sociodemographic and health characteristics. Several attempts were made to contact all individuals by phone to confirm eligibility and willingness to participate. During recruitment, both the information letters and recruitment interviews conducted by trained personnel emphasized that participation was encouraged irrespective of the presence or absence of shoulder symptoms. Volunteers with contraindications to MRI, including prior shoulder replacement surgery, were excluded.

### Variables and Data Sources

Data collection items are listed in the eMethods in Supplement 1. The clinical visit comprised a standardized, comprehensive assessment of medical history and shoulder symptoms through questionnaires and a structured interview. Participants were first asked, “Have you experienced shoulder symptoms lasting more than 24 hours during the past week (either persistent or intermittent)?” Based on their responses, individuals were classified as asymptomatic (answering no) or symptomatic (answering yes). Those reporting current symptoms were further asked to identify the affected side. The history of previous shoulder symptoms was obtained from all participants. Shoulder pain and function were evaluated using the Shoulder Pain and Disability Index,^28^ Constant score,^29^ and Subjective Shoulder Value.^30^ Finally, a structured clinical examination was conducted by shoulder and elbow surgeons with more than 10 years of experience (T.I., R.B., A.R., K.K., V.L., A.J., and L.R).

After clinical data collection, participants underwent bilateral shoulder MRI using 3T scanners of similar model and configuration (Siemens Magnetom Skyra and Vida Fit; Siemens Healthineers) equipped with dedicated phased-array shoulder coils. The imaging sequences and parameters are detailed in the eMethods in Supplement 1. No intra-articular contrast enhancement was used. Before the formal interpretation of the images, a study radiologist (N.S., F.B., and V.H.) screened each participant’s scans for incidental pathologies—such as tumors or infections—that might require urgent diagnostic evaluation and medical intervention.

The formal evaluation of the MRI images was conducted by 3 radiologists with more than 10 years of experience in musculoskeletal imaging each. Each scan was independently reviewed by ^2^ of the 3 radiologists (N.S., F.B., and V.H.) using standardized assessment forms shown in the eMethods in Supplement 1. Radiologists were blinded to demographic information and patient history, results of the clinical examination, as well as to each other’s readings. Any disagreements were resolved by consensus.

Each of the 4 RC tendons was evaluated separately and graded according to the Zlatkin classification.^31^ Signal increase and/or inhomogeneity on fluid-sensitive sequences was considered indicative of tendinopathy. A fluid-filled defect of the tendon tissue extending to either surface or the tendon insertion on more than 2 consecutive images was interpreted as a PTT. To qualify as an FTT, a defect had to extend to both surfaces of the tendon, connecting the subacromial space and glenohumeral joint.

We defined RC abnormality as tendinopathy, PTT, or FTT of any of the RC tendons. MRI findings for each shoulder were classified based on the most severe abnormality observed in any of the 4 RC tendons, using an ordinal severity scale: FTT (most severe), followed by PTT, tendinopathy, and normal tendon. The highest-grade abnormality among the tendons was recorded as the representative finding for that shoulder. For per-person prevalence estimates, the shoulder with the more severe abnormality was used. In addition, we assessed all images for abnormalities of the glenohumeral and acromioclavicular joints and long head of the biceps tendons, as outlined in the eMethods in Supplement 1.

### Statistical Analysis

All statistical analyses were conducted using R statistical software version 4.4.3 (R Foundation for Statistical Computing). Significance was set at 2-sided *P* < .05. Details of the statistical methods can be found in the eMethods in Supplement 1.

Participant characteristics were summarized using demographic and clinical information. Means and SDs were reported for continuous variables and proportions for categorical variables. These characteristics were also analyzed by symptom status: current symptoms, prior symptoms only, or no history of shoulder symptoms. We compared the prevalence of imaging abnormalities between currently asymptomatic and symptomatic shoulders and between shoulders with and without a history of prior shoulder symptoms, injuries, or surgeries. Differences between groups were analyzed using Fisher exact test or the Wilcoxon rank sum test, as appropriate.

Interobserver agreement for MRI findings was assessed using both Cohen κ, Gwet AC2, and percentage agreement,^32^ with ordinal weights applied to the 4-category outcomes. Analyses were performed using the *iccCAC* package.

To minimize potential selection bias, we applied inverse probability (IP) weighting to account for nonparticipation in the FIMAGE study. Covariates for study participation included registry-based variables (available for the full Health 2000 survey sample) and survey-based variables from the Health 2000 and 2011 survey participants.^26,33^ Missing data among these variables due to item nonresponse were addressed using multiple imputation via the *mice* package, using the random forest method to generate 50 imputed datasets. For each imputed dataset, IP weights were estimated using random forest models, and the final weights were calculated as the average across all imputations.

Because imaging was performed bilaterally, shoulders were considered clustered within individuals, and analyses of shoulder-level data accounted for this intraindividual correlation. Missing data due to item nonresponse were addressed by creating 50 imputed datasets using the random forest method. In addition to the FIMAGE study variables, all variables used in imputing missing values for the IP weighted dataset were included. The sampling design, IP weights, and multiple imputations were accounted for in estimating prevalences and analyzing regression models using the *survey* package.

Differences in the prevalence of MRI-detected abnormalities between asymptomatic and symptomatic participants were analyzed using multinomial logistic regression models. Three sequential models were constructed. Model 1 adjusted for age, sex, level of education, and geographic region. Model 2 additionally accounted for other MRI-detected shoulder abnormalities, because shoulder pain may not always originate from the RC tendons. Multiple structures—such as glenohumeral or acromioclavicular osteoarthritis and biceps tendon pathology—can independently contribute to symptoms. Including these variables in the model helped account for potential confounding from non-RC sources of pain and isolate the association of interest. Model 3 adjusted for abnormal clinical shoulder tests because clinicians often emphasize that treatment decisions are not based on imaging alone. Including pain-provocation and strength-deficit tests allowed us to examine whether combining clinical and imaging findings improves discrimination between symptomatic and asymptomatic shoulders. Details of the radiological and clinical covariates are provided in the eMethods in Supplement 1. We also compared the prevalence of imaging abnormalities between shoulders with and without a history of prior shoulder symptoms in a sensitivity analysis adjusted for age, sex, level of education, and geographic region.

Adjusted prevalences by symptom group were estimated using predictive margins derived from the multinomial models. Adjusted prevalence differences were interpreted as the difference in outcome prevalence between groups after controlling for potential confounders, providing easier interpretation of the association between shoulder symptoms and RC abnormalities.

## Results

### Characteristics of the Study Population

**Figure 1.**
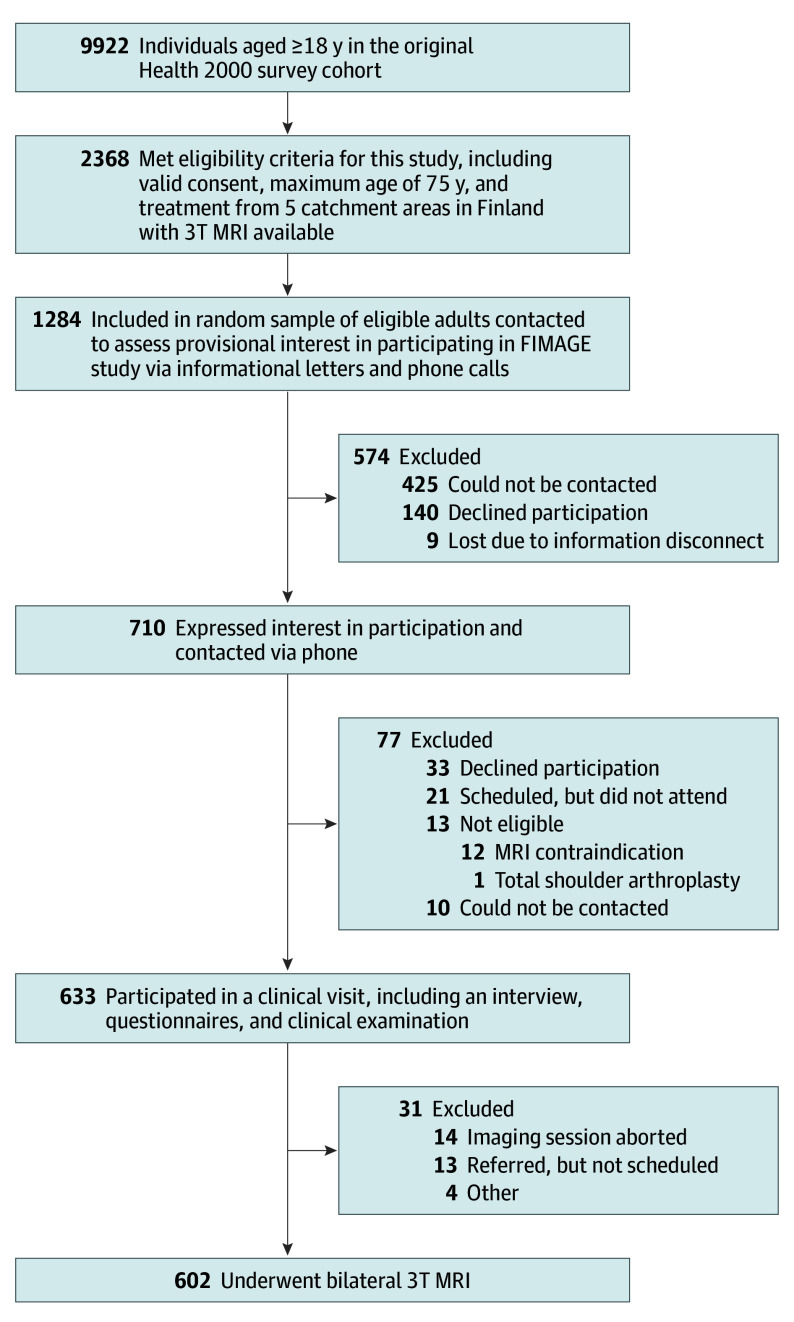
Recruitment Flowchart of the FIMAGE Study FIMAGE indicates Finnish Imaging of Shoulder; MRI, magnetic resonance imaging; 3T, 3-Tesla.

A total of 602 participants (median age, 58 [range, 41-76] years) underwent clinical shoulder examination and bilateral shoulder MRI and were included in the study. Of these, 313 (52.0%) were females and 289 (48.0%) were males. At the time of the research visit, 110 participants (18%) reported current shoulder symptoms. Among the asymptomatic group, 294 participants (60%) reported a previous history of shoulder symptoms (Table 1). Figure 1 shows the participant flow through recruitment, eligibility, and inclusion.

**Table 1.** Characteristics of the Study Participants by the Presence of Current Shoulder Symptoms^a^

Characteristics	All participants (N = 602), No. (%)	Participants by current symptom status, No. (%)		
		Asymptomatic (n = 492)	Symptomatic (n = 110)	SMD
Sex				
Female	313 (52.0)	256 (52.0)	57 (51.8)	0.00
Male	289 (48.0)	236 (48.0)	53 (48.2)	0.00
Current smoker	90 (15.0)	71 (14.4)	19 (17.3)	0.08
Right-hand dominance^b^	571 (94.9)	466 (94.7)	105 (95.5)	0.03
Employment (currently working)	376 (62.5)	316 (64.2)	60 (54.5)	0.20
Education (Bachelor’s degree or higher)	246 (40.9)	211 (42.9)	35 (31.8)	0.23
No previous shoulder symptoms^c^	198 (32.9)	198 (40.2)	0	1.16
Previous shoulder injuries^d^	162 (26.9)	121 (24.6)	41 (37.3)	0.28
Previous shoulder surgery^e^	55 (9.1)	36 (7.3)	19 (17.3)	0.31
Age, mean (SD), y	58.3 (9.7)	58.1 (9.7)	59.5 (9.5)	0.15
BMI, mean (SD)	27.6 (4.8)	27.6 (4.7)	27.8 (5.4)	0.04
SPADI, mean (SD)^f^^,^^g^	9.1 (15.0)	5.1 (10.4)	27 (18.9)	1.44
SPADI pain subscale, mean (SD)^g^	14 (20.4)	8 (14.3)	40.8 (22.2)	1.76
SPADI function subscale, mean (SD)^g^	6.1 (12.8)	3.3 (8.9)	18.4 (19.0)	1.02
Pain at night, mean (SD)^g^^,^^h^	0.6 (1.9)	0 (0.1)	3.4 (3.1)	1.55
Pain at rest in the daytime, mean (SD)^g^^,^^h^	0.3 (1.2)	0 (0.2)	1.8 (2.2)	1.14
Pain with activity, mean (SD)^g^^,^^h^	1 (2.3)	0 (0.6)	5.0 (2.8)	2.44
Constant score, mean (SD)^i^^,^^j^	85.5 (11.6)	88.1 (9.1)	73.7 (14.0)	1.22
SSV, mean (SD)^j^^,^^k^	81.8 (17.0)	85.3 (14.8)	66.4 (18.0)	1.15

Abbreviations: BMI, body mass index (calculated as weight in kilograms divided by height in meters squared); SMD, standardized mean difference; SPADI, Shoulder Pain and Disability Index; SSV, Subjective Shoulder Value.

^a^ Intermittent or continuous shoulder symptoms lasting at least 1 day during the past week.

^b^ Self-reported; right-handed, left-handed, ambidextrous. Ambidextrous (n = 8) was classified as right-handed in the later analyses.

^c^ Participants reporting no previous shoulder symptoms.

^d^ Participants reporting any injury (sudden onset of symptoms) to either shoulder.

^e^ Participants reporting 1 or more shoulder surgeries to either side.

^f^ Range 0 to 100, where 0 is no pain and normal function.

^g^ Worst (higher) score of right and left side selected.

^h^ Range of 0 to 10, where 0 is no pain.

^i^ Range of 0 to 100, where 100 is no pain and normal function.

^j^ Worst (lower) score of right and left side selected.

^k^ Range of 0% to 100%, where 100% is normal shoulder.

### Population Prevalence of Imaging Abnormalities

RC abnormalities on MRI were found in 595 participants (98.7%; 95% CI, 97.5%-99.5%); of these, 25% had tendinopathy, 62% had PTTs, and 11% had FTTs (Table 2). Abnormalities were most common in the supraspinatus tendon (590 [98%]), followed by the infraspinatus (517 [86%]), subscapularis (499 [83%]), and teres minor (68 [11%]) tendons (eTable 1 in Supplement 1). Gwet AC2 values ranged from 0.86 to 0.96 across individual RC tendons, reflecting almost perfect interobserver agreement (eTable 2 in Supplement 1). Structural damage (presence of a PTT or an FTT) was slightly more common in the dominant shoulder. Of the 70 participants with FTTs, 26 (37%) had bilateral tears (eTable 3 in Supplement 1).

**Table 2.** Overall Prevalence of Rotator Cuff Abnormalities Per Participant (Either Shoulder; N = 602) in the Finnish Population Aged 41 to 76 Years^a^

Rotator cuff finding on MRI	Participants, No.	Prevalence, % (95% CI)
Normal	7	1.3 (0.5-2.5)
Tendinopathy	152	25.3 (21.7-29.1)
Partial-thickness tear	373	62.4 (58.2-66.5)
Full-thickness tear	70	11.1 (8.6-13.8)

Abbreviation: MRI, magnetic resonance imaging.

^a^ MRI findings for each shoulder were classified according to the most severe abnormality detected in any tendon. For the per-person prevalence calculation, the more severely affected shoulder was used.

### Population Prevalence of RC Abnormalities by Age

**Figure 2.**
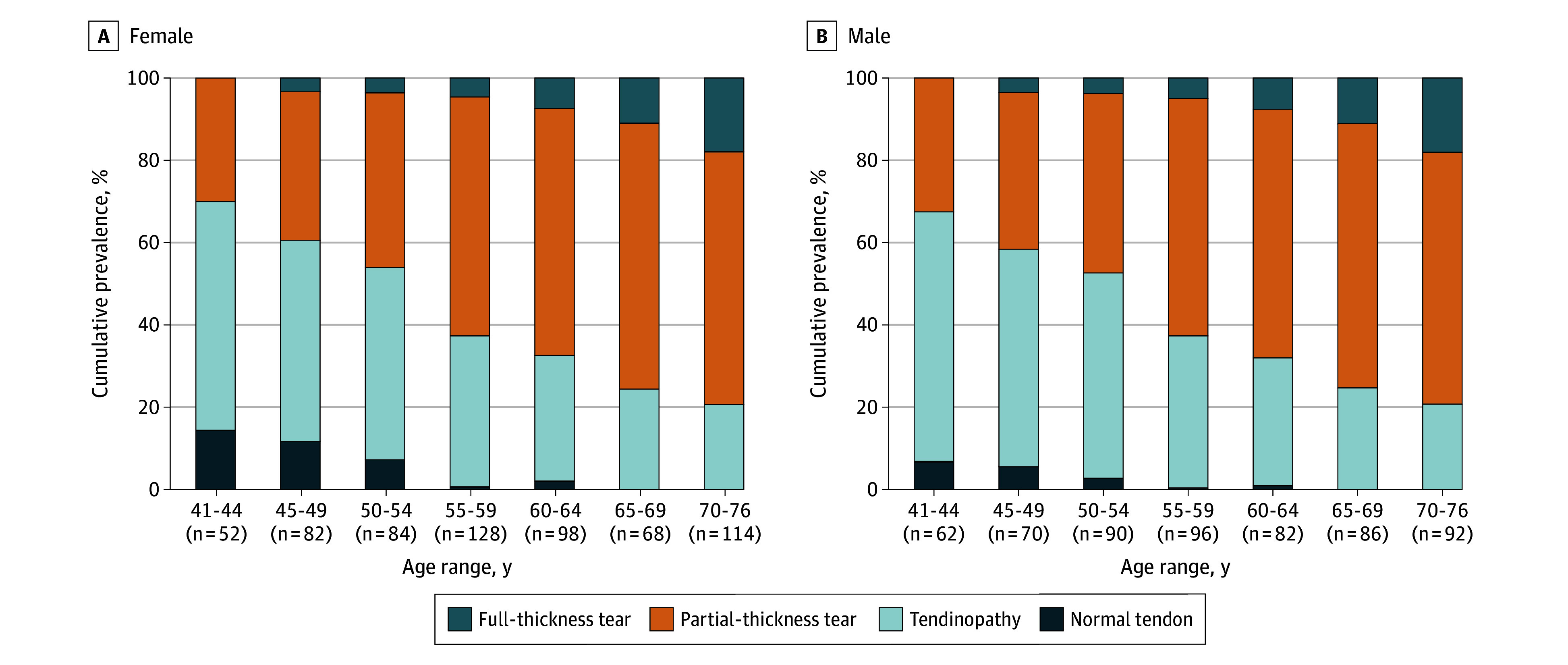
Bar Graph Showing the Prevalence of Rotator Cuff Abnormalities by Age and Sex

RC abnormalities showed a clear age-related progression, with milder findings in younger participants and more advanced structural damage in older participants (Figure 2). Tendinopathy was the most common finding in those aged 40 to 54 years, whereas structural tendon damage (PTT or FTT) was the predominant abnormality from age 55 years and older. No FTTs were observed in individuals younger than 45 years, but prevalence increased from 4% in the group aged 45 to 49 years to 28% in those aged 70 years and older. The prevalence of abnormalities was similar between males and females (eTable 4 in Supplement 1).

### Population Prevalence of RC Abnormalities in Asymptomatic and Symptomatic Shoulders

The differences in sociodemographic and clinical characteristics of asymptomatic (492 [81.7%]) and symptomatic (110 [18.3%]) participants are summarized in Table 1. Those reporting current shoulder symptoms had moderate pain and disability. They were more likely to have a history of shoulder pain, injuries, and surgeries compared with currently asymptomatic individuals. Among currently asymptomatic participants, sociodemographic and clinical differences as measured by percentage and mean (SD) between those with and without a history of shoulder symptoms were small (eTable 5 in Supplement 1).

Of the 1204 shoulders, 1076 (90.6%) were asymptomatic and 128 (10.4%) were symptomatic. RC abnormalities were observed in 96% of asymptomatic shoulders (1039 of 1076) and 98% of symptomatic shoulders (126 of 128), with a prevalence difference of 1.8% (95% CI, −2.9% to 4.7%). While the prevalence of tendinopathy and PTTs did not differ between symptomatic and asymptomatic shoulders, FTTs were more common in symptomatic shoulders (14.6%) than in asymptomatic shoulders (6.5%), yielding a prevalence difference of 8.1% (95% CI, 1.8%-15.1%) (Table 3). Among the 96 FTTs observed, 75 (78%) were identified in asymptomatic shoulders. Of the 26 participants with bilateral FTTs, 17 (65%) reported no symptoms in either shoulder and 5 (19%) reported symptoms in only 1 shoulder (eFigure in Supplement 1).

**Table 3.** Prevalence of Rotator Cuff Abnormalities on MRI Per Shoulder According to the Absence or Presence of Current Shoulder Symptoms in 1204 Shoulders and Differences in Prevalence Adjusted by Clinically Relevant Confounders^a^

Rotator cuff finding on MRI	Asymptomatic (n = 1076)		Symptomatic (n = 128)		Difference in prevalence, % (95% CI)^c^		
	Patients, No.	Prevalence, % (95% CI)^b^	Patients, No.	Prevalence, % (95% CI)^b^	Model 1: population prevalence^b^	Model 2: plus other imaging abnormalities^d^	Model 3: plus clinical rotator cuff tests^e^
Normal	37	3.9 (2.4 to 5.7)	2	2.1 (0.0 to 6.2)	−1.8 (−4.7 to 2.9)	−1.8 (−4.8 to 2.9)	−2.5 (−5.2 to 2.0)
Tendinopathy	427	38.9 (35.2 to 42.2)	34	30.4 (22.7 to 39.4)	−8.4 (−17.3 to 1.1)	−7.8 (−16.9 to 2.1)	−3.3 (−12.9 to 7.7)
Partial-thickness tear	537	50.6 (46.9 to 54.3)	71	52.8 (42.2 to 61.8)	2.1 (−8.8 to 12.8)	2.9 (−7.5 to 13.2)	5.0 (−6.0 to 16.0)
Full-thickness tear	75	6.5 (4.9 to 8.3)	21	14.6 (8.8 to 20.4)	8.1 (1.8 to 15.1)	6.7 (1.4 to 12.2)	0.8 (−3.4 to 6.0)

Abbreviation: MRI, magnetic resonance imaging.

^a^ MRI findings for each shoulder were classified according to the most severe abnormality detected in any tendon. Intermittent or continuous shoulder symptoms were those that lasted at least 1 day during the past week.

^b^ Adjusted for age, sex, education, and region.

^c^ The difference in prevalence is calculated as the prevalence in symptomatic minus prevalence in asymptomatic participants.

^d^ Adjusted for age, sex, education, region, and coexisting MRI-detected shoulder abnormalities.

^e^ Adjusted for age, sex, education, region, coexisting MRI-detected shoulder abnormalities, and positive clinical rotator cuff tests.

After adjusting for the 2 clinically relevant potential confounders—presence of imaging abnormalities in other shoulder structures and positive clinical RC tests—the difference in FTT prevalence between asymptomatic and symptomatic participants was no longer observed (model 3: difference, 0.8%; 95% CI, −3.4% to 6.0%) (Table 3). Also, accounting for tear size did not change the result in model 3 (eTable 6 in Supplement 1). We also compared the prevalence of imaging abnormalities between shoulders with (418) and without (658) a history of prior shoulder symptoms. Overall, prevalence patterns were similar between groups, with the exception of FTTs, which were more frequent in previously symptomatic shoulders (absolute difference, 4.3%; 95% CI, 0.8%-8.1%) (eTable 7 in Supplement 1).

## Discussion

In a representative sample of the general Finnish population aged 41 to 76 years, nearly all individuals (99%) had at least 1 RC abnormality detected on MRI. The prevalence and severity of these findings increased with age and no substantial differences were observed between sexes. PTTs were present in 43% of participants aged 45 years or younger, increasing to 65% in those aged 70 years or older, while FTTs increased from 0% to 29%. Most RC abnormalities were detected in asymptomatic individuals, highlighting the poor concordance between shoulder symptoms and imaging abnormalities.

The high prevalence of abnormal MRI findings and their poor concordance with symptoms challenges the routine attribution of shoulder symptoms directly to imaging abnormalities. In our study, both tendinopathy and PTTs were equally common in symptomatic and asymptomatic shoulders. While FTTs were more prevalent in symptomatic shoulders in unadjusted analyses, there was no difference between symptomatic and asymptomatic shoulders after adjusting for clinically relevant confounders, such as clinical examination findings and concurrent MRI abnormalities. While we cannot dismiss the possibility that some RC tears may contribute to shoulder symptoms, our findings indicate that we are currently unable to distinguish clinically meaningful MRI abnormalities from incidental findings. This limitation persists even when using state-of-the-art 3T MRI and conducting detailed clinical assessments by experienced shoulder specialists, highlighting the limited value of imaging and clinical tests for diagnosing RC disorders and guiding treatment decisions.

These findings have important implications for test interpretation in routine clinical care. In middle-aged and older adults, the extremely high pretest probability of MRI-detected RC changes—approaching 100% in individuals over age 50—means that the mere presence of an abnormality has limited diagnostic value. Such high pretest probability reduces the specificity and positive-predictive value of MRI findings in detecting a clinically meaningful condition, making it essential to shift from asking whether abnormalities exist to whether they plausibly explain the clinical findings. This reasoning applies even to FTTs because, especially with increasing age, the baseline likelihood of detecting such tears is substantial, and most are asymptomatic. Consequently, a positive MRI result does not confirm causality unless features such as a clear traumatic event, acute strength loss, or persistent functional deficit increase the pretest probability. This approach minimizes anchoring on incidental findings and supports proportionate, patient-centered care.

RC abnormalities were detected in nearly all shoulders of participants older than age 40 years, challenging the use of traditional terms such as *tear*. While we refer to these findings as abnormalities, many likely represent normal age-related changes rather than clinically relevant structural changes. Adopting more precise and less value-laden terminology—such as lesion, defect, fraying, disruption, structural alteration, or degeneration—may help reduce patient anxiety and the perceived need to do something or fix something by avoiding language that implies trauma or a requirement for repair. At a health-system level, clearer language could reduce overdiagnosis and overtreatment, conserving resources for higher value care.

The existing literature on the prevalence of abnormal RC imaging findings is limited. To our knowledge, only 3 prior studies, all at high risk of bias, have reported population-based prevalence estimates for RC tears, all of which are broadly consistent with our findings.^24^ A South Australian MRI study (30; 60% female; aged 56-74 years) found a 43% prevalence of PTTs and 20% prevalence of FTTs.^34^ An ultrasound study from North London (463; 100% female; aged 64-78 years) reported a PTT prevalence of 34% and an FTT prevalence of 11%.^35^ Another ultrasound-based study consisting of the population of a single mountain village in Japan (683; 67% female; aged 22-87 years) found an FTT prevalence of 17%.^36^ A systematic review of 56 studies found no consistent association between imaging findings and symptoms, with most studies being of low methodological quality and showing conflicting results.^22^

### Limitations

Some limitations of this study warrant consideration. First, concerns may arise regarding whether noncontrast MRI provides sufficient accuracy for detecting partial or small RC tears compared with magnetic resonance arthrography (MRA). However, 2 systematic reviews found that MRI performed comparably to MRA.^37,38^ Given the invasive nature of MRA, it is less suitable for population-based research, supporting standard MRI as a more feasible and ethically acceptable modality for this study. Second, because our sample was population-based rather than drawn from individuals actively seeking care, it likely reflects a milder disease spectrum than is typically seen in specialty care, where patients may present with more pronounced symptoms and larger RC tears. In our supplementary analysis, an increase in tear size did not influence the difference in prevalence between asymptomatic and symptomatic shoulders in the fully adjusted model. However, large tears were uncommon in our cohort, limiting our ability to evaluate this subgroup, and conclusions regarding more severe cases should therefore be made with caution. Third, the possibility of selection bias cannot be ruled out. To evaluate this, we compared the final study sample with the original, nationally representative Health 2000 population across key demographic characteristics and found no notable differences. For example, the observed 7-day prevalence of shoulder pain in the original Health 2000 survey (a general health survey without a specific focus on musculoskeletal complaints) was 17%, closely matching the 18% reported in our current study sample.^2^ Relevant confounders were also accounted for in the statistical analyses. Fourth, the age range of our participants (41-76 years) limits the generalizability of our findings to younger adults, in whom RC abnormalities are more likely to result from acute traumatic events rather than degenerative changes. Finally, fluency in Finnish or Swedish was a requirement for participation in the study, which may have led to limited ethnic diversity within the sample and should be considered when generalizing our findings to other populations.

## Conclusions

In this cross-sectional study, MRI examination of the shoulder found that RC abnormalities are present in nearly all individuals over 40 years of age, irrespective of symptoms. Given that tendinopathy, PTTs, and even FTTs may be incidental findings, clinicians should consider their high population prevalence when interpreting imaging results and deciding on interventions targeting these abnormalities. Reframing many of these findings as normal age-related changes rather than disease may help guide more appropriate care and reduce unnecessary interventions. In this context, adopting more precise and less value-laden terminology may help avoid language that implicitly suggests that something is broken and requires fixing. Such terminology may reduce patient anxiety, discourage unnecessary surgical interventions, and help minimize overdiagnosis and overtreatment.

## Notes

Supplement 1.
eMethods
**eFigure.** Distribution of symptoms in participants with bilateral full-thickness tears **eTable 1.** Crude prevalence of MRI abnormalities per tendon, per dominant and non-dominant shoulder and per person **eTable 2.** Interobserver agreement statistics: Cohen’s kappa, Gwet’s AC2 and percent agreement, with ordinal weights applied to the four-category outcomes **eTable 3.** Distribution of full-thickness tears (FTT) per affected tendon and per shoulder **eTable 4.** Prevalence of MRI abnormalities by sex **eTable 5.** Sociodemographic and clinical differences among participants according to no, past, and current shoulder symptoms **eTable 6.** Adjusted differences in prevalence of full thickness rotator cuff tears in MRI per shoulder according to the absence or presence of current shoulder symptoms and size of the tear in 1204 shoulders by clinically relevant confounders **eTable 7.** Prevalence of rotator cuff abnormalities in MRI per shoulder according to the absence or presence of current and previous shoulder symptoms in 1204 shoulders and differences in prevalence
eReferences

Supplement 2.
Data Sharing Statement

## References

[1] Luime JJ, Koes BW, Hendriksen IJ, . Prevalence and incidence of shoulder pain in the general population; a systematic review. Scand J Rheumatol. 2004;33(2):73-81. doi:10.1080/03009740310004667 15163107

[2] Kaila-Kangas L, ed. Musculoskeletal disorders and diseases in Finland: results of the Health 2000 survey. The National Public Health Institute; 2007. Accessed December 19, 2025. https://www.julkari.fi/server/api/core/bitstreams/969b2990-3936-41b1-a469-062e573f4d56/content

[3] Koskinen S, Lundqvist A, Ristiluoma N. Health, Functional Capacity and Welfare in Finland in 2011. Finnish Institute for Health and Welfare; 2012.

[4] Urwin M, Symmons D, Allison T, . Estimating the burden of musculoskeletal disorders in the community: the comparative prevalence of symptoms at different anatomical sites, and the relation to social deprivation. Ann Rheum Dis. 1998;57(11):649-655. doi:10.1136/ard.57.11.649 9924205 PMC1752494

[5] Adamson J, Ebrahim S, Dieppe P, Hunt K. Prevalence and risk factors for joint pain among men and women in the West of Scotland Twenty-07 study. Ann Rheum Dis. 2006;65(4):520-524. doi:10.1136/ard.2005.037317 16126799 PMC1798081

[6] Ostör AJ, Richards CA, Prevost AT, Speed CA, Hazleman BL. Diagnosis and relation to general health of shoulder disorders presenting to primary care. Rheumatology (Oxford). 2005;44(6):800-805. doi:10.1093/rheumatology/keh598 15769790

[7] van der Windt DA, Koes BW, de Jong BA, Bouter LM. Shoulder disorders in general practice: incidence, patient characteristics, and management. Ann Rheum Dis. 1995;54(12):959-964. doi:10.1136/ard.54.12.959 8546527 PMC1010060

[8] Haas R, Gorelik A, O’Connor DA, Pearce C, Mazza D, Buchbinder R. Patterns of imaging requests by general practitioners for people with musculoskeletal complaints: an analysis from a primary care database. Arthritis Care Res (Hoboken). 2025;77(3):402-411. doi:10.1002/acr.25189 37403274 PMC11848978

[9] Cuff A, Parton S, Tyer R, Dikomitis L, Foster N, Littlewood C. Guidelines for the use of diagnostic imaging in musculoskeletal pain conditions affecting the lower back, knee and shoulder: a scoping review. Musculoskeletal Care. 2020;18(4):546-554. doi:10.1002/msc.1497 32755058

[10] Ottenheijm RP, Cals JW, Winkens B, Weijers RE, de Bie RA, Dinant GJ. Ultrasound imaging to tailor the treatment of acute shoulder pain: a randomised controlled trial in general practice. BMJ Open. 2016;6(11):e011048. doi:10.1136/bmjopen-2016-011048 27872111 PMC5128954

[11] Buchbinder R, Staples MP, Shanahan EM, Roos JF. General practitioner management of shoulder pain in comparison with rheumatologist expectation of care and best evidence: an Australian national survey. PLoS One. 2013;8(4):e61243. doi:10.1371/journal.pone.0061243 23613818 PMC3628939

[12] Awerbuch MS. The clinical utility of ultrasonography for rotator cuff disease, shoulder impingement syndrome and subacromial bursitis. Med J Aust. 2008;188(1):50-53. doi:10.5694/j.1326-5377.2008.tb01507.x 18205566

[13] Australian Government Services Australia. Medicare Item Reports. 2025. Accessed May 15, 2025. https://medicarestatistics.humanservices.gov.au/statistics/mbs_item.html

[14] Colvin AC, Egorova N, Harrison AK, Moskowitz A, Flatow EL. National trends in rotator cuff repair. J Bone Joint Surg Am. 2012;94(3):227-233. doi:10.2106/JBJS.J.00739 22298054 PMC3262185

[15] Vidal C, Lira MJ, de Marinis R, Liendo R, Contreras JJ. Increasing incidence of rotator cuff surgery: A nationwide registry study in Chile. BMC Musculoskelet Disord. 2021;22(1):1052. doi:10.1186/s12891-021-04938-7 34930197 PMC8690465

[16] Judge A, Murphy RJ, Maxwell R, Arden NK, Carr AJ. Temporal trends and geographical variation in the use of subacromial decompression and rotator cuff repair of the shoulder in England. Bone Joint J. 2014;96-B(1):70-74. doi:10.1302/0301-620X.96B1.32556 24395314

[17] Englund M, Guermazi A, Gale D, . Incidental meniscal findings on knee MRI in middle-aged and elderly persons. N Engl J Med. 2008;359(11):1108-1115. doi:10.1056/NEJMoa0800777 18784100 PMC2897006

[18] Jensen MC, Brant-Zawadzki MN, Obuchowski N, Modic MT, Malkasian D, Ross JS. Magnetic resonance imaging of the lumbar spine in people without back pain. N Engl J Med. 1994;331(2):69-73. doi:10.1056/NEJM199407143310201 8208267

[19] Jaeschke R, Guyatt GH, Sackett DL. Users’ guides to the medical literature. III. How to use an article about a diagnostic test. B. What are the results and will they help me in caring for my patients? The Evidence-Based Medicine Working Group. JAMA. 1994;271(9):703-707. doi:10.1001/jama.1994.03510330081039 8309035

[20] Ibounig T, Rämö L, Haas R, . Imaging abnormalities of the acromioclavicular joint and subacromial space are common in asymptomatic shoulders: a systematic review. J Orthop Surg Res. 2025;20(1):7. doi:10.1186/s13018-024-05378-4 39754140 PMC11697641

[21] Ibounig T, Sanders S, Haas R, . Systematic Review of Shoulder Imaging Abnormalities in Asymptomatic Adult Shoulders (SCRUTINY): abnormalities of the glenohumeral joint. Osteoarthritis Cartilage. 2024;32(10):1184-1196. doi:10.1016/j.joca.2024.06.001 38876437

[22] Tran G, Cowling P, Smith T, . What imaging-detected pathologies are associated with shoulder symptoms and their persistence? A systematic literature review. Arthritis Care Res (Hoboken). 2018;70(8):1169-1184. doi:10.1002/acr.23554 29513925 PMC6099421

[23] Teunis T, Lubberts B, Reilly BT, Ring D. A systematic review and pooled analysis of the prevalence of rotator cuff disease with increasing age. J Shoulder Elbow Surg. 2014;23(12):1913-1921. doi:10.1016/j.jse.2014.08.001 25441568

[24] Sanders S, Ibounig T, Haas R, . Rotator cuff imaging abnormalities in asymptomatic shoulders: a systematic review. J Orthop Sports Phys Ther. 2025;55(12):1-16. doi:10.2519/jospt.2025.13611 41308021

[25] Ibounig T, Buchbinder R, Sillanpää N, ; FIMAGE investigators. Concordance of shoulder symptoms and imaging findings: a protocol for the Finnish Imaging of Shoulder (FIMAGE) study. BMJ Open. 2023;13(12):e074457. doi:10.1136/bmjopen-2023-074457 38154899 PMC10759117

[26] Heistaro S. Methodology report: Health 2000 survey. National Public Health Institute; 2008. Accessed December 19, 2025. https://www.julkari.fi/server/api/core/bitstreams/eff82691-7cc7-4836-aa0a-0ee9dbed3d52/content

[27] Aromaa A, Koskinen S. Health and functional capacity in Finland: baseline results of the Health 2000 health examination survey. 2004. Accessed December 19, 2025. https://www.julkari.fi/server/api/core/bitstreams/a2d594ee-4c2a-4861-bd4b-df3776fd7542/content

[28] Roach KE, Budiman-Mak E, Songsiridej N, Lertratanakul Y. Development of a shoulder pain and disability index. Arthritis Care Res. 1991;4(4):143-149. doi:10.1002/art.1790040403 11188601

[29] Constant CR, Murley AH. A clinical method of functional assessment of the shoulder. Clin Orthop Relat Res. 1987;(214):160-164. doi:10.1097/00003086-198701000-00023 3791738

[30] Gilbart MK, Gerber C. Comparison of the subjective shoulder value and the Constant score. J Shoulder Elbow Surg. 2007;16(6):717-721. doi:10.1016/j.jse.2007.02.123 18061114

[31] Zlatkin MB, Iannotti JP, Roberts MC, . Rotator cuff tears: diagnostic performance of MR imaging. Radiology. 1989;172(1):223-229. doi:10.1148/radiology.172.1.2740508 2740508

[32] Gwet KL. Computing inter-rater reliability and its variance in the presence of high agreement. Br J Math Stat Psychol. 2008;61(Pt 1):29-48. doi:10.1348/000711006X126600 18482474

[33] Lundqvist A, Mäki-Opas T. Health 2011 survey—methods. The National Institute for Health and Welfare; 2016. Accessed December 19, 2025. https://www.julkari.fi/server/api/core/bitstreams/e9dd181a-e46b-4fdf-9812-6311d2eb5d4f/content

[34] Gill TK, Shanahan EM, Allison D, Alcorn D, Hill CL. Prevalence of abnormalities on shoulder MRI in symptomatic and asymptomatic older adults. Int J Rheum Dis. 2014;17(8):863-871. doi:10.1111/1756-185X.12476 25294682

[35] Hinsley H, Ganderton C, Arden NK, Carr AJ. Prevalence of rotator cuff tendon tears and symptoms in a Chingford general population cohort, and the resultant impact on UK health services: a cross-sectional observational study. BMJ Open. 2022;12(9):e059175. doi:10.1136/bmjopen-2021-059175 36100305 PMC9472112

[36] Yamamoto A, Takagishi K, Osawa T, . Prevalence and risk factors of a rotator cuff tear in the general population. J Shoulder Elbow Surg. 2010;19(1):116-120. doi:10.1016/j.jse.2009.04.006 19540777

[37] Liu F, Cheng X, Dong J, Zhou D, Han S, Yang Y. Comparison of MRI and MRA for the diagnosis of rotator cuff tears: a meta-analysis. Medicine (Baltimore). 2020;99(12):e19579. doi:10.1097/MD.0000000000019579 32195972 PMC7220562

[38] Lenza M, Buchbinder R, Takwoingi Y, Johnston RV, Hanchard NC, Faloppa F. Magnetic resonance imaging, magnetic resonance arthrography and ultrasonography for assessing rotator cuff tears in people with shoulder pain for whom surgery is being considered. Cochrane Database Syst Rev. 2013;2013(9):CD009020. doi:10.1002/14651858.CD009020.pub2 24065456 PMC6464715

