# Supplementary material for: Incidental Rotator Cuff Abnormalities on Magnetic Resonance Imaging
Source: JAMA Intern Med. 2026 Feb 16;186(4):406–14. doi: 10.1001/jamainternmed.2025.7903 (PMC12910452; doi:10.1001/jamainternmed.2025.7903)
Supplement: Supplement 1. — eMethods eFigure. Distribution of symptoms in participants with bilateral full-thickness tears eTable 1. Crude prevalence of MRI abnormalities per tendon, per dominant and non-dominant shoulder and per person eTable 2. Interobserver agreement statistics: Cohen’s kappa, Gwet’s AC2 and percent agreement, with ordinal weights applied to the four-category outcomes eTable 3. Distribution of full-thickness tears (FTT) per affected tendon and per shoulder eTable 4. Prevalence of MRI abnormalities by sex eTable 5. Sociodemographic and clinical differences among participants according to no, past, and current shoulder symptoms eTable 6. Adjusted differences in prevalence of full thickness rotator cuff tears in MRI per shoulder according to the absence or presence of current shoulder symptoms and size of the tear in 1204 shoulders by clinically relevant confounders eTable 7. Prevalence of rotator cuff abnormalities in MRI per shoulder according to the absence or presence of current and previous shoulder symptoms in 1204 shoulders and differences in prevalence eReferences [file jamainternmed-e257903-s001.pdf]

Supplemental Online Content

Ibounig T, Järvinen TLN, Raatikainen S, et al. Incidental rotator cuff abnormalities on magnetic resonance imaging. *JAMA Intern Med*. Published online February 16, 2026. doi:10.1001/jamainternmed.2025.7903

eMethods
eFigure. Distribution of symptoms in participants with bilateral full-thickness tears
eTable 1. Crude prevalence of MRI abnormalities per tendon, per dominant and non-dominant shoulder and per person
eTable 2. Interobserver agreement statistics: Cohen's kappa, Gwet's AC2 and percent agreement, with ordinal weights applied to the four-category outcomes
eTable 3. Distribution of full-thickness tears (FTT) per affected tendon and per shoulder
eTable 4. Prevalence of MRI abnormalities by sex
eTable 5. Sociodemographic and clinical differences among participants according to no, past, and current shoulder symptoms
eTable 6. Adjusted differences in prevalence of full thickness rotator cuff tears in MRI per shoulder according to the absence or presence of current shoulder symptoms and size of the tear in 1204 shoulders by clinically relevant confounders
eTable 7. Prevalence of rotator cuff abnormalities in MRI per shoulder according to the absence or presence of current and previous shoulder symptoms in 1204 shoulders and differences in prevalence
eReferences

This supplemental material has been provided by the authors to give readers additional information about their work.

© 2026 Ibounig T et al. *JAMA Internal Medicine*.

eMethods

Study Population

Participants for the FIMAGE study were randomly selected among participants of the Health 2000 survey. Utilizing the Health 2000 study as our baseline sample provides rich longitudinal data on a wide range of health, sociodemographic, and behavioral factors, allowing us to correct for potential selection bias due to nonparticipation in the subsequent FIMAGE study. Detailed background information is available from at least two time points (2000 and 2023–2024), and for many participants, from three time points, as a large proportion also took part in the intermediate Health 2011 survey.

Health 2000 survey

The Health 2000 survey was carried out from fall 2000 to spring 2001 under the leadership of the National Public Health Institute (currently known as the National Institute for Health and Welfare, THL). Data collection included extensive face-to-face health interviews, self-administered questionnaires, laboratory and functional capacity tests, comprehensive clinical health examinations, and linkage to national health registries.[1-3] A representative sample of the Finnish adult population was obtained using a two-stage stratified cluster sampling design.

Initially, Finland was divided into five university hospital regions, each encompassing approximately one million inhabitants. Within each region, 16 health care districts were selected as clusters. Of the 80 health care districts nationwide, the 15 largest were selected with certainty (probability = 1), with sample sizes proportional to the population sizes. The remaining 65 districts were selected using systematic probability proportional to size (PPS) sampling within each stratum. To ensure proportional representation across regions, equal sample sizes were drawn from the smaller districts within each university hospital region. The final Health 2000 sample consisted of 9,922 individuals

© 2026 Ibounig T et al. *JAMA Internal Medicine*.

aged 18 years or older. To ensure adequate representation of older adults, individuals aged 80 years and older were over-sampled by doubling the sampling fraction.

Health 2011 survey

A follow-up study, the Health 2011 survey, was conducted ten years later, from fall 2011 to spring 2012.[4,5] All surviving participants from the original Health 2000 survey were invited to participate, and a total of 5,903 individuals completed the Health 2011 follow-up assessment. By 2011, all original participants were at least 29 years old. To refresh the cohort, a new random sample of individuals aged 18–28 years was drawn. Additionally, participants from the earlier Mini-Finland survey (conducted between 1978 and 1980) who were still alive and had also participated in the Health 2000 study (n = 1,278 from seven regions) were invited to take part in the Health 2011 survey.

FIMAGE Study Sample and Representativeness

The FIMAGE study sample was drawn from the Health 2000 cohort, and therefore inherits all advantages and limitations of this national sampling frame. Although no observational study can be completely free from selection bias, several procedures were implemented during recruitment and analysis to evaluate and strengthen representativeness. These procedures complement the information provided in the main manuscript and the sections describing statistical analyses, missing data, and the use of inverse probability weights with multiple imputation.

© 2026 Ibounig T et al. *JAMA Internal Medicine*.

Population-Based Sampling Frame

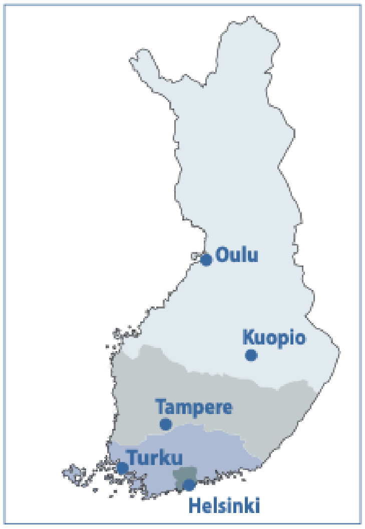

The original Health 2000 cohort achieved exceptionally high participation rates (89% for interviews and 85% for clinical examinations), making this cohort one of the most representative population-based samples internationally. The present FIMAGE study recruited a random sub-sample of Health 2000 participants living within a reasonable traveling distance to one of Finland's five university hospitals. Recruitment areas included both metropolitan and rural regions. A map published in our study protocol illustrates the location of these cities and the population distribution: most Finns reside in the southern part of the country, and the highlighted regions represent areas where approximately one-quarter of the population lives.

Recruitment Monitoring and Interim Assessment of Representativeness

Early in recruitment, and in collaboration with experts from THL, we conducted an interim comparison after enrolling the first 200 participants. Using national registry data, we compared sociodemographic and health-related characteristics of our sample (FIMAGE study) with those of the full Health 2000 cohort. We were prepared to enrich recruitment if clear deviations emerged. Importantly, no major differences were observed in baseline characteristics, including known or suspected risk factors for rotator cuff tears. We therefore proceeded, with the expectation that any remaining small imbalances could be corrected analytically.

Adjustment for Nonresponse and Attrition Using Inverse Probability Weighting

Given the time elapsed between the original Health 2000 survey and the recruitment of the FIMAGE cohort, some degree of attrition-related selection was expected. To mitigate potential bias arising

© 2026 Ibounig T et al. *JAMA Internal Medicine*.

from differential participation, inverse probability weighting (IPW) was applied. The method estimates each individual's probability of participation based on a wide range of sociodemographic and health characteristics available from high-quality national registries for both participants and nonparticipants. Each participant then receives a statistical weight inversely proportional to this estimated probability. Individuals who were less likely to participate—for example due to younger age, lower socioeconomic status, or higher morbidity—are given greater weight in the analysis, whereas those who were more likely to participate contribute proportionally less.

The effectiveness of IPW depends critically on the availability of comprehensive and accurate predictors of participation. In this study, we had exceptionally detailed registry data for both participants and nonparticipants covering variables such as variables including age, sex, region, educational level, employment status, sickness absence, disability benefits, chronic diseases, and healthcare utilization. Because we could model participation probability using these data, IPW effectively balanced the sample on key characteristics that matter most for representativeness.

Evaluation of Potential Symptom-Related Selection Bias

Given the focus on shoulder conditions, we also considered the possibility that individuals with shoulder symptoms might be more inclined to participate. This risk was addressed in two ways. First, participant information letters and telephone communications emphasized that participation was important regardless of the presence or absence of shoulder symptom status. Second, we evaluated empirical evidence for such bias by comparing symptom prevalence in the FIMAGE sample with those from the original Health 2000 survey and published literature:

- The 7-day prevalence of shoulder pain in the original Health 2000 survey was 17%[6], closely matching the 18% observed in our current sample.
- Published estimates of lifetime prevalence of shoulder pain reach up to 67%[7], and 1-year prevalence can be as high as 55%[8]. Our observed lifetime prevalence of 60% aligns well with these benchmarks.

© 2026 Ibounig T et al. *JAMA Internal Medicine*.

These comparisons suggest that our sample does not exhibit a strong bias toward individuals with symptomatic shoulders.

Limitations

The only aspect we could not account for was individuals aged 41 and older who immigrated to Finland after 2000. We acknowledge this limitation; however, their proportion in this age group remains small and is unlikely to meaningfully affect representativeness.

Given these measures, we believe our analytical sample remains as close to a nationally representative, population-based sample as is feasible within the constraints of observational research.

Clinical Data Collection Items

**Interview**	
	History of current and previous shoulder symptoms
	Previous shoulder injuries and surgeries
	History of sports or hobbies with shoulder targeted loads
	Multisite pain
	Worry about pain and fear avoidance
	Work history and exposures
**Questionnaires**	
**Shoulder specific**	SPADI
	Constant Score
	Subjective Shoulder Value
**Other validated questionnaires**	PCS-6 (Pain Catastrofizing Scale, 6-item)
	PSEQ (Pain Self Efficacy Questionnaire)
	HADS (Hospital Anxiety and Depression scale)
	EQ-5D-5L (Quality of Life)
	SILS (Single Item Literacy Screener)
**Other items**	Demographics
	Medical history
	History of shoulder and neck symptoms
	Lifestyle (incl. physical activity, smoking, alcohol consumption, sleep, HRQoL)
**Range of Motion and Strength Measurements**	
**Active and passive shoulder ROM**	Flexion
	Abduction
	External rotation in 90 degree abduction
	Internal rotation in 90 degree abduction
**Active shoulder movements**	External rotation, arm by the side

© 2026 Ibounig T et al. *JAMA Internal Medicine*.

	Hand behind neck reach
	Hand behind back reach
**Shoulder strength**	Abduction, arm by the side
	Abduction at 90 degrees, scapular plane
	External rotation, arm by the side
	Internal rotation, arm by the side
	Internal rotation, belly press position
**Other**	Scapular rotation during active abduction
	Thoracic kyphosis
**Special Shoulder Tests**	
**Stability**	Apprehension
	Relocation
	Jerk test
**Subacromial shoulder pain**	Painful arch
	Hawkings-Kennedy
**Rotator cuff tests**	Drop arm (SSP)
	Full can (SSP)
	Empty can/Jobe (SSP)
	Zero-degree abduction test (SSP)
	External rotation lag sign (SSP, ISP)
	Drop sign (ISP)
	5th finger test (ISP)
	Resisted External Rotation Test (ISP)
	Hornblower / Patte sign (ISP, TM)
	Lift off (SSC)
	Internal Rotation Lag Sign (SSC)
	Belly-press (SSC)
	Bear Hug (SSC)
**AC-joint**	AC-joint pain in internal rotation
	High painful arch
	Cross-body adduction
	O'Brian / Active compression test
**Long head of biceps and superior labrum**	Speeds (LHB)
	Yergason (LHB)
	O'Brian (LHB, SLAP)

Abbreviations: SPADI = Shoulder Pain and Disability Index, HRQoL = Health-related quality of life, ROM = Range of motion, SSP = Supraspinatus, ISP = Infraspinatus, TM = Teres minor, SSC = Subscapularis, AC = Acromioclavicular, LHB = Long head of biceps, SLAP = Superior labrum anterior and posterior

© 2026 Ibounig T et al. *JAMA Internal Medicine*.

MRI Sequences and Imaging Parameters

Imaging device: 3.0 Tesla Siemens Magnetom Skyra and Vida Fit

Sequence	Orientation	TR (ms)	TE (ms)	FOV (mm)	Matrix Size (%)	Slice Thickness / Gap (mm)
PDfs	Sagittal	4000–5500	30	160×160	384×80	3.0 / 0.3
PDfs	Axial	4000–5500	35	140×140	320×80	2.5 / 0.25
PDfs	Coronal	4000–5500	35	140×140	320×80	2.5 / 0.5
T2	Sagittal	4000–5500	100	140×140	384×80	3.0 / 0.3
T1	Sagittal	828	27	160×160	320×80	3.0 / 0.3
T1 Dixon-VIBE	Axial	6.68	2.46	250×250	352×90	1.5–1.8

Abbreviations: TR = repetition time; TE = echo time; FOV = field of view; PDfs = proton density fat-saturated; VIBE = volumetric interpolated breath-hold examination.

© 2026 Ibounig T et al. *JAMA Internal Medicine.*

Imaging Data Collection Items

Outline of the complete set of radiological assessment forms used in the FIMAGE study for both X-ray and MRI imaging.

X-ray	
**AC Joint**	Osteoarthritis (Abreu classification, Grade 0–3)
**Subacromial space**	Grade 1–4
	Minimum height (mm)
**Calcific tendinosis**	Bosworth classification (Grade 0–3)
**GH Joint**	Osteoarthritis (Guyette classification, Grade 1–4)
	Osteoarthritis (Samilson Prieto Allain classification 0–4)
**Scapular anatomy**	Acromial spur (Yes / No)
	Acromion (Bigliani classification 1–3)
	Os acromiale (Yes / No)
	Critical Shoulder Angle (degrees)
	Acromion Index (mm)
	Acromion slope (degrees)
	Lateral Acromion angle (degrees)
**Other**	Hill-Sachs lesion (Yes / No)
	Bony Bankart lesions (Yes / No)
	Status post proximal fracture (Yes / No)
	Osteonecrosis (Yes / No)
MRI (3T)	
**AC Joint**	Osteoarthritis (Abreu classification, Grade 0–3)
	Effusion and / or synovitis (Yes / No)
	Bone edema (Yes / No)
**Subacromial space (bursa)**	Bursitis (Grade 1–4)
**Rotator cuff**	Zlatkin classification (Grade 0–3)
	Tendinosis (Grade 0–2)
	Tear thickness (% Score, 0–4)
	AP tear size (Score 0–3) (If full-thickness tear)
	AP tear width (mm) (If full-thickness tear)
	Longitudinal tear length (mm) (If full-thickness tear)
	Tendon retraction length (mm) (If full-thickness tear)
	Calcifications (Yes / No); AND (if Yes) Largest concrement (mm)
**Rotator cuff muscles**	Muscle atrophy (Goutallier classification 0–4)
	Traumatic muscle edema (Yes / No)
	Dixon quantitative fat analysis (%)
	3D muscle volume (mm^3/cm^3)
**Nerves**	Denervation edema (Yes / No)
	Suprascapular notch entrapment (Yes / No)
**GH Joint (Bone and cartilage)**	Hill-Sachs lesion (Yes / No); AND (if Yes) Largest dimension (mm)
	Bony Bankart lesions (Yes / No); AND (if Yes) Dislocation (maximum), Fragment height (mm), Fragment width (mm)
	Other fractures (Yes / No)
	Osteonecrosis (Yes / No)
**GH Joint (degeneration)**	Cartilage (Grade 1–3)

© 2026 Ibounig T et al. *JAMA Internal Medicine*.

	Subchondral bone cyst (Yes / No)
	Bone edema (Yes / No)
**Biceps and Labrum**	Anterior labrum (Grade 1–3)
	Posterior labrum (Grade 1–3)
	Labral cysts (anterior AND posterior) (Yes / No)
	Long head of biceps (Grade 0–3)
	SLAP (Snyder classification 0–4)
	Joint capsule (Grade 1–3)
**Scapular anatomy**	Acromial spur (Yes / No)
	Acromion (Bigliani classification 1–3)
	Posterior glenoid retroversion (degrees)
	Critical Shoulder Angle (degrees)
	Acromion Index (mm)
	Acromion slope (degrees)
	Lateral Acromion angle (degrees)
**Anatomical variants**	Sublabral recess (Yes / No)
	Sublabral hole (Yes / No)
	Bufort complex (Yes / No)
	Os acromiale (Yes / No)
	Bare area, glenoid center (Yes / No)
	Bare area, posterolateral humeral head (ISP) (Yes / No)
	Bare area, lateral humeral head (SSP) (Yes / No)
	Tubercle of Assaki (Yes / No)

Abbreviations: AC = Acromioclavicular, GH = Glenohumeral, SLAP = Superior labrum anterior and posterior, ISP = Infraspinatus, SSP = Supraspinatus

© 2026 Ibounig T et al. *JAMA Internal Medicine*.

Assessment of coexisting MRI-Detected Shoulder Abnormalities

We assessed all MR images for abnormalities of the glenohumeral (GH) and acromioclavicular (AC) joints, and the long head of the biceps tendon (LHBT).

Structure	Classification	Radiological assessment
Glenohumeral joint	0, Normal cartilage<br>1, Damaged cartilage<br>2, End stage OA	A focal defect in the articular cartilage filled with non-cartilage material was considered to represent damaged cartilage along with general, frank thinning of the articular cartilage with some concomitant subchondral reactive changes (bone marrow lesion or cyst). End stage OA was present if the cartilage damage was extensive and combined with subchondral reactive changes and deformation of the subchondral bone.
Acromioclavicular joint	0, No arthritis<br>1, Mild arthritis<br>2, Moderate arthritis<br>3, Severe arthritis	**Abreu classification: Grade 1** AC joint OA consisted of joint space narrowing, and/or irregularity of the joint margins, and/or presence of high signal in T2-weighted images in the joint (last criteria assessed only with MR imaging); **Grade 2** consisted of Grade 1 plus the presence of subchondral cysts, and/or bone sclerosis, and/or small osteophytes <2 mm, and/or soft tissue formation <2 mm around the joint (last criteria only assessed with MR imaging); and **Grade 3** consisted of Grade 2 plus the presence of large osteophytes >2 mm, and/or soft tissue proliferation >2 mm, and/or mass effect on the RC beneath the AC joint (the last two criteria assessed only with MR imaging). Mass effect was defined as deformity of the muscle or tendon contour. An osteophyte was considered present if an osseous excrescence greater than 1 mm projected from the clavicle or acromion, or both, with radiography or with sagittal or coronal T1-weighted imaging.
Long head of biceps tendon	0, Normal<br>1, Tendinopathy<br>2, Partial tear<br>3, Full thickness tear	Signal increase or inhomogeneity on fluid sensitive sequences was considered indicative of tendinopathy. A fluid filled defect of the tendon tissue extending to either surface of the tendon insertion on more than two consecutive images was interpreted as a partial tear. A full-thickness tear required a defect to extend through the whole substance of the tendon.

Abbreviations: MR = Magnetic resonance, OA = Osteoarthritis, RC = Rotator cuff

© 2026 Ibounig T et al. *JAMA Internal Medicine*.

Statistical Methods

The R statistical software were utilized in all analyses.[9]

Missing Data

Inverse probability weights with multiple imputation

The primary method to correct for the effects of nonparticipation was based on inverse probability weights (IPW).[10] Predictors of participation in the bilateral MRI included

- Register variables available for all sample members (area, age, sex, hospital and primary health care visits for diagnoses related to shoulder symptoms categorized as never, more than two years and within two years before the FIMAGE study), and
- Survey data from the Health 2000 and 2011 Surveys (education, smoking, leisure-time physical activity, problems with the work environment/physical workload causing harm to coping at work, BMI, blood pressure, cholesterol) available for the survey participants.[1,5]

Due to item nonresponse, some of the predictors of the nonparticipation contained missing values, and we used multiple imputation (MI) to fill these.[11,12] We applied the mice package to create 50 imputed datasets using the random forest method and the predictors listed above.[13]

For each imputed dataset, the IPWs were calculated using the random forest method.[14,15] The most important predictors were BMI, cholesterol, age, systolic blood pressure, work environment/physical workload, diastolic blood pressure, smoking, education, area and leisure time physical exercise in terms of mean decrease in node impurity.

The resulting weights for the analyses were taken as the average over the imputed datasets.

© 2026 Ibounig T et al. *JAMA Internal Medicine*.

Statistical Analyses

Inter-Rater Reliability

We applied both Cohen's kappa and Gwet's AC2 using the ordinal weights for the four-category outcomes to assess the inter-rater agreement using the iccCAC package.[16,17] For binary outcomes, it has been noted, that Gwet's AC1 is often larger than Cohen's kappa, and Gwet's AC1 can be high in the case of the minimal possible agreement.[18]

Descriptive statistics

Background characteristics of the participants were reported using means and standard deviations for continuous variables, and prevalences for categorical variables. These figures were reported also in subgroups defined by the presence of current, previous, or no history of shoulder symptoms.

Multiple imputation

MI was conducted separately for the analysis dataset. All variables, which were used in imputing the missing values of the IPW dataset, were included, and also the FIMAGE study variables, which were used in these analyses. Also here, 50 imputed datasets were created using the random forest method.

Analyses representing the Finnish population

We applied the survey package of the R statistical software to implement the stratified sampling design, the IPW and MI.[19] Clustering was accounted for in analyses of shoulder-specific characteristics. Weights were applied in all analyses below, and additionally multiple imputation in case of item-nonresponse in the analysis variables.

Regression analyses

We applied multinomial logistic regression models. Model 1 contained age, sex, education and region for adjustment. Model 2 contained model 1 and other shoulder degeneration in MRI. Model 3 contained model 2 and pathological findings in rotator cuff clinical examination.

© 2026 Ibounig T et al. *JAMA Internal Medicine*.

Adjusted prevalences

We calculated predictive margins based on a multinomial logistic regression model to calculate the adjusted prevalences in groups defined by the symptom status.[20] We used bootstrap with 200 resamples in each imputed datasets (10,000 in total) to obtain the 95% confidence intervals.[19]

© 2026 Ibounig T et al. *JAMA Internal Medicine*.

Radiological and Clinical Covariates

This supplement describes the grading and reclassification methods used for imaging findings and clinical shoulder tests. *Imaging findings* were initially graded using an ordinal severity scale with either three or four levels, depending on the anatomical structure. For analysis purposes, two thresholds were applied to distinguish between normal and abnormal findings: a higher diagnostic threshold, which included only moderate to severe imaging abnormalities, and a lower threshold, which also classified mild abnormalities as abnormal. Similarly, *clinical shoulder tests* were originally scored using an ordinal scale with either three or four response options. Two reclassification approaches were used to define positive versus negative test results: one considered only positive findings from functional rotator cuff tests, while the other included positive findings from both functional tests and pain provocation tests. The test battery was designed to be both methodologically rigorous and reflective of current clinical practice, drawing on tests commonly used by shoulder surgeons[21] and those with the highest diagnostic accuracy as identified in systematic reviews and meta-analyses.[22-26]

<table>
<tr><th>Imaged structure</th><th>Original classification</th><th>Method of reclassification in analyses</th><th>Re-classification</th><th>Covariate</th></tr>
<tr><td></td><td></td><td></td><td></td><td>Mild imaging findings</td></tr>
<tr><td>GH Joint</td><td>0, Normal cartilage
1, Damaged cartilage
2, End stage OA</td><td rowspan="3">Any finding greater than grade 0 was considered abnormal. Grade 0 was classified as normal, whereas grades 1 (mild), 2 (moderate), and 3 (severe) were classified as abnormal.</td><td>0, Normal
1, Abnormal</td><td rowspan="3">0, No structural abnormalities
1, Abnormality in one anatomical structure
2, Abnormalities in two (or more) anatomical structures</td></tr>
<tr><td>AC Joint</td><td>0, No arthritis
1, Mild arthritis
2, Moderate arthritis
3, Severe arthritis</td><td>0, Normal
1, Abnormal</td></tr>
<tr><td>Long head of biceps</td><td>0, Normal
1, Tendinopathy
2, Partial tear
3, Full thickness tear</td><td>0, Normal
1, Abnormal</td></tr>
<tr><td></td><td></td><td></td><td></td><td>Severe imaging findings</td></tr>
<tr><td>GH Joint</td><td>0, Normal cartilage
1, Damaged cartilage
2, End stage OA</td><td rowspan="3">Only moderate or severe changes were considered abnormal. Grades 0 (normal) and 1 (mild) were classified as normal, whereas grades 2 (moderate) and 3 (severe) were considered abnormal.</td><td>0, Normal
1, Abnormal</td><td rowspan="3">0, No structural abnormalities
1, Abnormality in one anatomical structure
2, Abnormalities in two (or more) anatomical structures</td></tr>
<tr><td>AC Joint</td><td>0, No arthritis
1, Mild arthritis
2, Moderate arthritis
3, Severe arthritis</td><td>0, Normal
1, Abnormal</td></tr>
<tr><td>Long head of biceps</td><td>0, Normal
1, Tendinopathy
2, Partial tear
3, Full thickness tear</td><td>0, Normal
1, Abnormal</td></tr>
</table>

© 2026 Ibounig T et al. *JAMA Internal Medicine.*

Clinical test	Original classification	Method of reclassification	Re-classification	Covariate
**SSP function**				**RC tests (only functional)**
External rotation lag sign	0, Negative<br>1, Positive<br>2, Test not possible	When test not possible (=2, due to pain or limited ROM) score interpreted as missing.	0, Negative<br>1, Positive	0, All tests negative<br>1, One test positive<br>2, Two or more tests positive
Abduction strength	0, Normal<br>1, Mild weakness<br>2, Severe weakness<br>3, Non-functional	Any grade of reduced strength classified as positive.	0, Negative<br>1, Positive	
Drop arm	0, Negative<br>1, Positive<br>2, Test not possible	When test not possible (=2, due to pain or limited ROM) score interpreted as missing.	0, Negative<br>1, Positive	
**ISP function**				
External rotation strength	0, Normal<br>1, Mild weakness<br>2, Severe weakness<br>3, Non-functional	Any grade of reduced strength classified as positive.	0, Negative<br>1, Positive	
External rotation lag sign	0, Negative<br>1, Positive<br>2, Test not possible	When test not possible (=2, due to pain or limited ROM) score interpreted as missing.	0, Negative<br>1, Positive	
5th finger test	0, Negative<br>1, Positive<br>2, Test not possible	When test not possible (=2, due to pain or limited ROM) score interpreted as missing.	0, Negative<br>1, Positive	
Hornblow	0, Negative<br>1, Positive<br>2, Test not possible	When test not possible (=2, due to pain or limited ROM) score interpreted as missing.	0, Negative<br>1, Positive	
**SSC function**				
Internal rotation strength	0, Normal<br>1, Mild weakness<br>2, Severe weakness<br>3, Non-functional	Any grade of reduced strength classified as positive.	0, Negative<br>1, Positive	
Lift-off test	0, Negative<br>1, Positive<br>2, Test not possible	When test not possible (=2, due to pain or limited ROM) score interpreted as missing.	0, Negative<br>1, Positive	
Internal rotation lag sign	0, Negative<br>1, Positive<br>2, Test not possible	When test not possible (=2, due to pain or limited ROM) score interpreted as missing.	0, Negative<br>1, Positive	
Belly press	0, Negative<br>1, Positive<br>2, Test not possible	When test not possible (=2, due to pain or limited ROM) score interpreted as missing.	0, Negative<br>1, Positive	
Bearhug	0, Negative<br>1, Positive<br>2, Test not possible	When test not possible (=2, due to pain or limited ROM) score interpreted as missing.	0, Negative<br>1, Positive	

© 2026 Ibounig T et al. *JAMA Internal Medicine*.

**TM function**				
Hornblow	0, Negative<br>1, Positive<br>2, Test not possible	When test not possible (=2, due to pain or limited ROM) score interpreted as missing.	0, Negative<br>1, Positive	
**Pain**				**RC tests (pain and function)**
Full can	0, Negative<br>1, Weakness<br>2, Pain<br>3, Test not possible	Weakness and pain combined as a positive result. Test not possible (=3) interpreted as missing.	0, Negative<br>1, Positive	0, All tests negative<br>1, One test positive<br>2, Two or more tests positive
Empty can	0, Negative<br>1, Weakness<br>2, Pain<br>3, Test not possible	Weakness and pain combined as a positive result. Test not possible (=3) interpreted as missing.	0, Negative<br>1, Positive	
Painful arch	0, Negative<br>1, Positive	NA	0, Negative<br>1, Positive	
Hawkins-Kennedy	0, Negative<br>1, Positive<br>2, Test not possible	When test not possible (=2, due to pain or limited ROM) score interpreted as missing.	0, Negative<br>1, Positive	

Abbreviations: GH = Glenohumeral, AC = Acromioclavicular, SSP = Supraspinatus, RC = Rotator cuff, ISP = Infraspinatus, SSC = Subscapularis, TM = Teres minor

© 2026 Ibounig T et al. *JAMA Internal Medicine*.

eFigure 1. Distribution of symptoms in participants with bilateral full-thickness tears (n = 26).

Despite having bilateral full-thickness tears, only 15% of individuals reported symptoms in both shoulders, highlighting a frequent mismatch between imaging findings and symptom presentation.

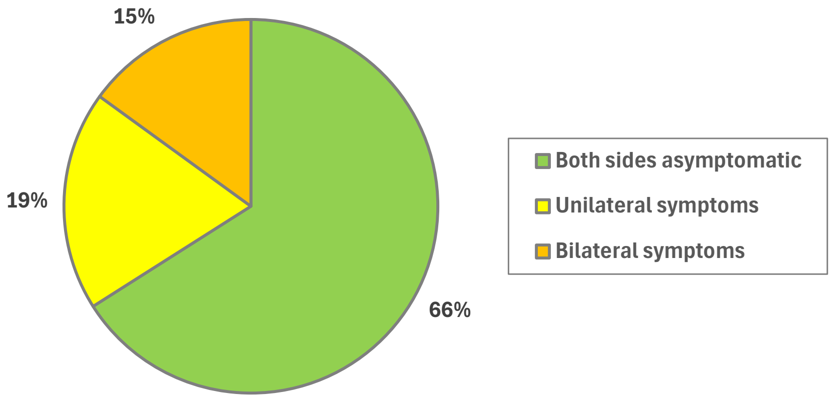

© 2026 Ibounig T et al. *JAMA Internal Medicine*.

eTable 1. Crude prevalence of MRI abnormalities per tendon, per dominant and non-dominant shoulder and per person.

Prevalence of rotator cuff findings among 602 participants						
	Dominant shoulder		Non-dominant shoulder		Person[a]	
	n	%	n	%	n	%
**RC (all tendons)**[b]						
Normal	16	3	23	4	7	1
Tendinopathy	214	36	247	41	152	25
Partial Tear	312	52	296	49	373	62
Full Tear	60	10	36	6	70	12
**SSP**						
Normal	25	4	34	6	12	2
Tendinopathy	256	43	298	50	206	34
Partial Tear	263	44	234	39	316	52
Full Tear	58	10	36	6	68	11
**ISP**						
Normal	133	22	184	31	85	14
Tendinopathy	368	61	360	60	388	64
Partial Tear	88	15	46	8	109	18
Full Tear	13	2	12	2	20	3
**SSC**						
Normal	142	24	202	34	103	17
Tendinopathy	318	53	287	48	307	51
Partial Tear	139	23	110	18	186	31
Full Tear	3	0.5	3	0.5	6	1
**TM**						
Normal	556	92	570	95	534	89
Tendinopathy	45	7	32	5	67	11
Partial Tear	1	0.2	0	0	1	0.2
Full Tear	0	0	0	0	0	0

Abbreviations: RC = Rotator cuff, SSP = Supraspinatus, ISP = Infraspinatus, SSC = Subscapularis, TM = Teres minor

[a] Findings of both shoulders combined and the worst finding of either shoulder selected.

[b] All tendons per shoulder combined and the worst finding per shoulder selected.

© 2026 Ibounig T et al. *JAMA Internal Medicine.*

eTable 2. Interobserver agreement statistics: Cohen's kappa[a], Gwet's AC2[b] and percent agreement[c], with ordinal weights applied to the four-category outcomes.

Structure	Measure	Coefficient	95% Confidence Interval
Supraspinatus tendon	Cohen's Kappa	0.69	0.65–0.72
	Gwet's AC2	0.89	0.88–0.90
	Percent Agreement	0.95	0.95–0.96
Infraspinatus tendon	Cohen's Kappa	0.60	0.56–0.65
	Gwet's AC2	0.88	0.87–0.89
	Percent Agreement	0.95	0.94–0.95
Subscapularis tendon	Cohen's Kappa	0.61	0.57–0.65
	Gwet's AC2	0.86	0.85–0.88
	Percent Agreement	0.94	0.94–0.95
Teres minor tendon	Cohen's Kappa	0.36	0.26–0.47
	Gwet's AC2	0.97	0.96–0.98
	Percent Agreement	0.97	0.97–0.98

[a] Cohen's Kappa (κ): Kappa adjusts for the level of agreement that could be expected by chance. However, kappa can be sensitive to the prevalence of findings and may underestimate agreement when categories are unbalanced.

[b] Gwet's AC2: This statistic also adjusts for chance agreement, like kappa, but is less affected by prevalence or marginal imbalance in ratings. It is considered more robust in cases with skewed distributions (e.g., when most ratings fall into a single category). Interpretation is similar to kappa, with higher values indicating stronger agreement.

[c] Percent Agreement: This is the simplest measure of agreement and refers to the percentage of times that two raters (e.g., radiologists) give the same rating. While easy to interpret, it does not account for agreement occurring by chance.

Interpretation of Agreement Scores

**Score Range**	**Interpretation**
0.00–0.20	Slight agreement
0.21–0.40	Fair agreement
0.41–0.60	Moderate agreement
0.61–0.80	Substantial agreement
0.81–1.00	Almost perfect agreement

© 2026 Ibounig T et al. *JAMA Internal Medicine.*

eTable 3. Distribution of full-thickness tears (FTT) per affected tendon and per shoulder.

A total of 125 full-thickness tears (FTTs) were identified in individual tendons, distributed across 96 shoulders. The majority were located in the supraspinatus (SSP) tendon, followed by the infraspinatus (ISP) and subscapularis (SSC) tendons. Of the 25 ISP tears, 23 (92%) occurred in shoulders with a concomitant SSP tear. All 6 SSC tears were also associated with an SSP tear. No full-thickness tears were observed in the teres minor tendon.

All FTTs were found in right-hand dominant participants. Of the 125 tears, 63 were unilateral and 31 were bilateral. The unilateral tears were observed in 44 individuals, and the bilateral tears in 26 individuals. All bilateral cases involved the SSP tendon, and 5 also included an ISP tear.

**Full thickness tears**	**Dominant (Right) Shoulder**	**Non-Dominant (Left) Shoulder**	**Bilateral[a] (Per Person)**
**SSP**	58	36	26
**ISP**	13	12	5
**SSC**	3	3	0
**FTTs per shoulder[b]**	60	36	26

[a] Number of individuals with a bilateral full thickness tear

[b] All tendons per shoulder combined (i.e. one or more FTTs per shoulder)

© 2026 Ibounig T et al. *JAMA Internal Medicine*.

eTable 4. Prevalence[a] of MRI abnormalities by sex

**Outcome**	**Level**	**no. (men)**	**Prevalence, men (%)**	**95% CI**	**no. (women)**	**Prevalence, women (%)**	**95% CI**
**RC all[b]**	Normal	1	0.4	0–1.6	6	2.2	0.8–4.4
	Tendinopathy	72	25.9	20.6–31.6	80	24.7	20–29.9
	Partial tear	183	62.6	56.4–68.6	190	62.2	56.4–67.7
	Full tear	33	11.2	7.7–15.4	37	11	7.7–14.8
**SSP[c]**	Normal	3	1.4	0.3–3.5	9	3.2	1.5–5.8
	Tendinopathy	108	38.3	32.2–44.5	98	29.8	24.7–35.3
	Partial tear	145	49.2	42.9–55.6	171	56.5	50.7–62.3
	Full tear	33	11.2	7.7–15.4	35	10.4	7.3–14.2
**ISP[c]**	Normal	41	15.1	11–19.9	44	13.7	10–18
	Tendinopathy	192	64.6	58.2–70.6	196	63.8	58–69.3
	Partial tear	46	16.5	11.9–22	63	19.6	15.2–24.5
	Full tear	10	3.8	1.8–6.7	10	3	1.5–5.2
**SSC[c]**	Normal	26	11	7.2–15.6	77	25.2	20.3–30.5
	Tendinopathy	159	53.8	47.4–60.2	148	47.5	41.6–53.5
	Partial tear	100	34.1	28.2–40.3	86	26.7	21.7–32.1
	Full tear	4	1.1	0.3–2.7	2	0.6	0.1–1.8
**TM[c]**	Normal	253	88.4	84.2–91.9	281	89.9	86–93.1
	Tendinopathy	35	11.3	7.9–15.5	32	10.1	6.9–14
	Partial tear	1	0.2	0–1.1	0	NA	NA
	Full tear	0	NA	NA	0	NA	NA

Abbreviations: RC = Rotator cuff, SSP = Supraspinatus, ISP = Infraspinatus, SSC = Subscapularis, TM = Teres minor, NA = Not applicable

[a] Adjusted for age, education and region.

[b] MRI findings for each shoulder were classified according to the most severe abnormality detected in any tendon. For the per-person prevalence calculation, the more severely affected shoulder was used.

[c] The more severely affected shoulder was used.

© 2026 Ibounig T et al. *JAMA Internal Medicine*.

eTable 5. Sociodemographic and clinical differences among participants according to no, past, and current shoulder symptoms

Characteristic	Asymptomatic (N=492)		Symptomatic
	No history of symptoms[a] (N=198)	Previous symptoms (N=294)	Current symptoms[b] (N=110)
	**n (%)**	**n (%)**	**n (%)**
Sex, female	109 (55)	146 (50)	57 (52)
Current smoker, n (%)	26 (13)	45 (15)	19 (17)
Right hand dominance[c], n (%)	190 (96)	276 (94)	105 (95)
Employment (currently working), n (%)	128 (65)	188 (64)	60 (55)
Previous shoulder injuries[d]	20 (10)	101 (34)	41 (37)
Previous shoulder surgery[e]	0 (0)	36 (12)	19 (17)
	**Mean (SD)**	**Mean (SD)**	**Mean (SD)**
Age (years), mean (SD)	57.8 (10.2)	58.3 (9.4)	59.5 (9.5)
Body-mass index, mean (SD)	27.8 (4.7)	27.4 (4.6)	27.8 (5.4)
Shoulder Pain and Disability Index (SPADI)[f,g]	2.6 (8.7)	6.7 (11.1)	27 (18.9)
SPADI pain subscale[g]	4 (11.4)	10.7 (15.4)	40.8 (22.2)
SPADI function subscale[g]	1.9 (7.6)	4.2 (9.6)	18.4 (19.0)
Pain at night[g,h]	0 (0)	0 (0.1)	3.4 (3.0)
Pain at rest in the daytime[g,h]	0 (0)	0 (0.2)	1.8 (2.2)
Pain with activity[g,h]	0 (0)	0.1 (0.7)	5.0 (2.8)
Constant Score[i,j]	89.4 (8.4)	87.2 (9.5)	73.7 (14)
Simple Shoulder Value (SSV)[j,k]	91 (12)	81 (95)	66 (18)

[a] Subjects reporting no previous shoulder symptoms

[b] Intermittent or continuous shoulder symptoms lasting at least one day during the past week

[c] Self-reported; right-handed, left-handed, ambidextrous. Ambidextrous (n=8) were classified as right-handed in the later analyses

[d] Subjects reporting any injury (sudden onset of symptoms) to either shoulder

[e] Subjects reporting one or more shoulder surgeries to either side

[f] Range 0 to 100, where 0 is no pain and no functional disability

[g] Worst (higher) score of right and left side selected

[h] Numerical rating scale 0 to 10, where 0 is no pain

[i] Range 0 to 100, where 100 is no pain and no functional disability

[j] Worst (lower) score of right and left side selected

[k] Range 0% to 100%, where 100% is subjectively normal shoulder

© 2026 Ibounig T et al. *JAMA Internal Medicine*.

eTable 6. Adjusted differences in prevalence[a] of full thickness rotator cuff tears in MRI per shoulder according to the absence or presence of current shoulder symptoms[b] and size of the tear in 1204 shoulders by clinically relevant confounders.

To further explore the association between rotator cuff (RC) abnormalities and shoulder symptoms, we conducted a supplementary analysis focusing specifically on full-thickness tears—the only category in the primary four-grade classification (normal tendon, tendinopathy, partial tear, full-thickness tear) that showed a notable difference in prevalence between symptomatic and asymptomatic shoulders. For this supplementary analysis, we applied a modified three-grade system: (1) no full-thickness tear, (2) a tear involving a single rotator cuff tendon, and (3) a tear involving two or more tendons. This refined classification was used to assess whether the extent of tendon involvement, as a proxy for tear size or severity, influenced prevalence rates and their association with symptoms. Due to the relatively small number of participants with full-thickness tears, point estimates of prevalence differences varied slightly between the primary and supplementary models. However, these differences were minor and fell well within the corresponding confidence intervals.

	Prevalence[c], % (95% CI)				Difference in prevalence[a], % (95% CI)		
**Outcome**	**N**	**Asymptomatic (n=1076)**	**N**	**Symptomatic (n=128)**	**Model 1[c]: Population prevalence**	**Model 2[d]: + Other imaging abnormalities**	**Model 3[e]: + Clinical RC tests**
One tendon	62	5.3 (4.0–6.9)	9	5.5 (2.3–9.6)	0.2 (−3.2–4.6)	0.1 (−3.3–4.3)	−1.5 (−4.5–1.8)
≥2 tendons	13	1.2 (0.6–1.8)	12	7.7 (4.0–11.9)	6.6 (2.8–10.9)	5.1 (1.5–9.2)	1.1 (−1.2–4.3)

[a] Difference in prevalence of full thickness rotator cuff tears between symptomatic and asymptomatic participants

[b] Intermittent or continuous shoulder symptoms lasting at least one day during the past week

[c] Adjusted for age, sex, education and region. (=population prevalence, including clustering effect)

[d] Adjusted for age, sex, education, region, clustering effect, and coexisting MRI-detected shoulder abnormalities. (= + other imaging abnormalities)

[e] Adjusted for age, sex, education, region, clustering effect, coexisting MRI-detected shoulder abnormalities, and positive clinical rotator cuff (RC) tests(= + clinical RC tests)

© 2026 Ibounig T et al. *JAMA Internal Medicine.*

eTable 7. Prevalence of rotator cuff abnormalities in MRI per shoulder[a] according to the absence or presence of current[b] and previous[c] shoulder symptoms in 1204 shoulders and differences in prevalence.

	Prevalence[d], % (95% CI)						Difference in prevalence[d], % (95% CI)		
**Rotator cuff finding in MRI**	**n**	**No history of symptoms (n=640)**	**n**	**Previous symptoms (n=436)**	**n**	**Current Symptoms (n=128)**	**Previous symptoms *minus* No history of symptoms**	**Current symptoms *minus* No history of symptoms**	**Current symptoms *minus* Previous symptoms**
Normal	28	4.4 (2.7 to 6.4)	9	3.0 (1.3 to 5.3)	2	2.1 (0.0 to 6.0)	−1.4 (−3.9 to 0.9)	−2.3 (−5.5 to 2.5)	−1.0 (−4.1 to 3.5)
Tendinopathy	274	40.7 (36.0 to 45.0)	153	36.2 (30.9 to 41.1)	34	30.4 (22.7 to 39.4)	−4.5 (−11.2 to 1.5)	−10.3 (−19.5 to 0.0)	−5.8 (−15.7 to 5.3)
Partial tear	319	50.1.0 (45.4 to 54.2)	218	51.6 (45.9 to 56.8)	71	52.9 (42.3 to 62.0)	1.5 (−4.8 to 7.6)	2.9 (−8.2 to 14.8)	1.4 (−9.9 to 12.8)
Full thickness tear	37	4.8 (3.1 to 6.7)	38	9.2 (6.1 to 12.8)	21	14.6 (8.7 to 20.4)	4.3 (0.8 to 8.1)	9.7 (3.6 to 16.9)	5.4 (−1.3 to 12.4)

[a] MRI findings for each shoulder were classified according to the most severe abnormality detected in any tendon.
[b] Intermittent or continuous shoulder symptoms lasting at least one day during the past week.
[c] Previous shoulder symptoms (self-reported), currently asymptomatic
[d] Adjusted for age, sex, education and region.

© 2026 Ibounig T et al. *JAMA Internal Medicine*.

Commented [TI1]: n=658
Commented [TI2]: n=418

eReferences

1. Heistaro S. Methodology report: Health 2000 Survey. . *National Public Health Institute* 2008; **B26/2008.** .
2. Aromaa A, Koskinen S. Health and functional capacity in Finland: Baseline results of the Health 2000 health examination survey. . *Kansanterveyslaitoksen julkaisuja B12/2004* 2004.
3. Kaila-Kangas L. Musculoskeletal disorders and diseases in Finland - Results of the Health 2000 survey. *Publications of the National Public Health Institute* 2007; (KTL B25/2007).
4. Koskinen S, Lundqvist A, Ristiluoma N. Health, functional capacity and welfare in Finland in 2011. *National Institute for Health and Welfare (THL)* 2012; **Report 68/2012**.
5. Lundqvist A, Mäki-Opas T. Health 2011 Survey - Methods. *The National Institute for Health and Welfare (THL)* 2016; **Raportti 8/2016.** .
6. Kaila-Kangas L. Musculoskeletal disorders and diseases in Finland - Results of the Health 2000 survey. Publications of the National Public Health Institute. Helsinki: Kansanterveyslaitos; 2007.
7. Luime JJ, Koes BW, Hendriksen IJ, et al. Prevalence and incidence of shoulder pain in the general population; a systematic review. *Scand J Rheumatol* 2004; **33**(2): 73–81.
8. Lucas J, van Doorn P, Hegedus E, Lewis J, van der Windt D. A systematic review of the global prevalence and incidence of shoulder pain. *BMC Musculoskelet Disord* 2022; **23**(1): 1073.
9. Team RC. R: A language and environment for statistical computing. Published online 2024: R Foundation for Statistical Computing, Vienna, Austria; 2024.
10. Robins JM, Andrea R, and Zhao LP. Estimation of Regression Coefficients When Some Regressors are not Always Observed. *Journal of the American Statistical Association* 1994; **89**(427): 846–66.
11. Rubin DB. Multiple Imputation for Nonresponse in Surveys. . New York.: John Wiley & Sons Inc.; 1987.
12. van Buuren S. Flexible Imputation of Missing Data. New York.: Chapman and Hall/CRC. ; 2018.
13. van Buuren S, Groothuis-Oudshoorn K. mice: Multivariate Imputation by Chained Equations in R. *Journal of Statistical Software* 2011; **45**(3): 1 – 67.
14. Breiman L, Friedman J, Olshen RA, Stone CJ. Classification and Regression Trees . 1st ed. ed. New York: Chapman and Hall/CRC; 1984.
15. Liaw A, Wiener M. Classification and regression by randomForest. *R news* 2002; **2**(3): 18–22.
16. Gwet KL. irrCAC: Computing Chance-Corrected Agreement Coefficients (CAC). . Published online 2019. ; 2019.
17. Gwet KL. Computing inter-rater reliability and its variance in the presence of high agreement. *Br J Math Stat Psychol* 2008; **61**(Pt 1): 29–48.

© 2026 Ibounig T et al. *JAMA Internal Medicine.*

18. Vach W, Gerke O. Gwet's AC1 is not a substitute for Cohen's kappa - A comparison of basic properties. *MethodsX* 2023; **10**: 102212.
19. Lumley T. Analysis of Complex Survey Samples. *Journal of Statistical Software* 2004; **9**(8): 1 – 19.
20. Graubard BI, Korn EL. Predictive margins with survey data. *Biometrics* 1999; **55**(2): 652–9.
21. Sciascia AD, Spigelman T, Kibler WB, Uhl TL. Frequency of use of clinical shoulder examination tests by experienced shoulder surgeons. *J Athl Train* 2012; **47**(4): 457–66.
22. Jain NB, Luz J, Higgins LD, et al. The Diagnostic Accuracy of Special Tests for Rotator Cuff Tear: The ROW Cohort Study. *Am J Phys Med Rehabil* 2017; **96**(3): 176–83.
23. Sgroi M, Loitsch T, Reichel H, Kappe T. Diagnostic Value of Clinical Tests for Supraspinatus Tendon Tears. *Arthroscopy* 2018; **34**(8): 2326–33.
24. Hanchard NC, Lenza M, Handoll HH, Takwoingi Y. Physical tests for shoulder impingements and local lesions of bursa, tendon or labrum that may accompany impingement. *Cochrane Database Syst Rev* 2013; **4**: CD007427.
25. Hermans J, Luime JJ, Meuffels DE, Reijman M, Simel DL, Bierma-Zeinstra SM. Does this patient with shoulder pain have rotator cuff disease?: The Rational Clinical Examination systematic review. *JAMA* 2013; **310**(8): 837–47.
26. Hegedus EJ, Goode AP, Cook CE, et al. Which physical examination tests provide clinicians with the most value when examining the shoulder? Update of a systematic review with meta-analysis of individual tests. *Br J Sports Med* 2012; **46**(14): 964–78.

© 2026 Ibounig T et al. *JAMA Internal Medicine*.
