# Supplementary material for: Incidental Rotator Cuff Abnormalities on Magnetic Resonance Imaging
Source: JAMA Intern Med. 2026 Feb 16;186(4):406–14. doi: 10.1001/jamainternmed.2025.7903 (PMC12910452; doi:10.1001/jamainternmed.2025.7903)
Supplement: Supplement 2. — Data Sharing Statement [file jamainternmed-e257903-s002.pdf]

Data Sharing Statement

Ibounig. Incidental Rotator Cuff Abnormalities on Magnetic Resonance Imaging. *JAMA Intern Med*. Published February 16, 2026. doi:10.1001/jamainternmed.2025.7903

Data
Data available: Yes
Data types: Deidentified participant data, Data dictionary
How to access data: De-identified participant-level data, software code, and the accompanying data dictionary may be shared in accordance with applicable European Union and Finnish legislation. Researchers with scientifically sound proposals should contact the corresponding author (thomas.ibounig@helsinki.fi). Following author review, data will be provided with investigator support after completion of a signed data protection agreement. Data will be available with publication until 5 years thereafter.
When available: With publication

Supporting Documents
Document types: Statistical/analytic code
How to access documents: De-identified participant-level data, software code, and the accompanying data dictionary may be shared in accordance with applicable European Union and Finnish legislation. Researchers with scientifically sound proposals should contact the corresponding author (thomas.ibounig@helsinki.fi). Following author review, data will be provided with investigator support after completion of a signed data protection agreement. Data will be available with publication until 5 years thereafter.
When available: With publication

Additional Information
Who can access the data: De-identified participant-level data, software code, and the accompanying data dictionary may be shared in accordance with applicable European Union and Finnish legislation. Researchers with scientifically sound proposals should contact the corresponding author (thomas.ibounig@helsinki.fi). Following author review, data will be provided with investigator support after completion of a signed data protection agreement. Data will be available with publication until 5 years thereafter.
Types of analyses: For any purpose.
Mechanisms of data availability: With investigator support.
Any additional restrictions: De-identified participant-level data, software code, and the accompanying data dictionary may be shared in accordance with applicable European Union and Finnish legislation. Researchers with scientifically sound proposals should contact the corresponding author (thomas.ibounig@helsinki.fi). Following author review, data will be provided with investigator support after completion of a signed data protection agreement. Data will be available with publication until 5 years thereafter.
